# An adjustable permeation membrane up to the separation for multicomponent gas mixture

**DOI:** 10.1038/s41598-019-43751-0

**Published:** 2019-05-14

**Authors:** Hongfei Ye, Dong Li, Xin Ye, Yonggang Zheng, Zhongqiang Zhang, Hongwu Zhang, Zhen Chen

**Affiliations:** 1 International Research Center for Computational Mechanics, State Key Laboratory of Structural Analysis for Industrial Equipment, Department of Engineering Mechanics, Faculty of Vehicle Engineering and Mechanics, Dalian University of Technology, Dalian, 116024 P.R. China; 2 Micro/Nano Science and Technology Center, Jiangsu University, Zhenjiang, 210013 P.R. China; 3 Department of Civil and Environmental Engineering, University of Missouri, Columbia, MO 65211 USA

**Keywords:** Carbon nanotubes and fullerenes, Computational nanotechnology

## Abstract

The mixture separation is of fundamental importance in the modern industry. The membrane-based separation technology has attracted considerable attention due to the high efficiency, low energy consumption, etc. However, the tradeoff between the permeability and selectivity is a crucial challenge, which is also difficult to adjust during the separation process. Based on the salt water-filled carbon nanotubes, a separation membrane with the adjustable molecular channels by the electric field is proposed in this work. The separation mechanism is clarified on the basis of the characteristic size of the molecular channel and the overall effective diameter of gas molecules. The molecular dynamics simulation is performed to examine the feasibility and validity of the designed separation membrane. The simulations on the binary gas mixture (H_2_ and N_2_) reveal the flow control and high-purity separation as the electric field intensity varies. As for the mixed gas with the three components (H_2_, N_2_ and Xe), the successive separations and the switch between the high-efficiency and high-purity separation could be achieved only through adjusting the electric field intensity. This work incorporates the control into the membrane-based separation technology, which provides a novel solution for the complex industrial separation requirement.

## Introduction

The mixture is the main existing form of the matter in nature. In the human life and industry, the mixture separation is a common and crucial means. It has wide applications in various fields, such as the water desalination^1^, wastewater purification^2,3^, natural gas processing^4^, oil refining^5^ and so on. Nowadays, there are numerous separation technologies available for the different types of the mixture, including solvent extraction^6^, distillation^7^, ultracentrifugation^8,9^, membrane separation^10^, etc. Among these methods, the membrane-based separation method has attracted considerable attention because of its high efficiency, low energy consumption and convenient operation^11^.

The membrane separation is referred to as a separation technology based on a selective membrane with a barrier to some components of the mixture. The separation process could be categorized in terms of the pore size as the microfiltration, ultrafiltration, nanofiltration and reverse osmosis^2^. In the recent decades, the membrane technology has been extensively applied in the gas separation^10,12,13^. Many relevant membrane materials for the gas separation are fabricated on the basis of the polymer^14–16^. Most of the efforts are focused on the enhancement of the gas permeability and selectivity through modifying the polymer membrane, e.g., the TiO_2_/polyimide nanocomposite membrane for the separation of H_2_ and N_2_^17^, the inorganic zeolite materials distributed in the organic polymer matrix^18,19^, incorporating metal-organic framework nanosheets into polymer matrices and polymeric membrane^20^, etc. With the advent of the nanoscale carbon materials, such as graphene and carbon nanotubes (CNTs), the carbon-based membrane is regarded as an ideal strategy to overcome the competition between the permeability and selectivity for gas separation^21,22^. Jiang *et al*. found the graphene sheets with functionalized pores have excellent selectivity far superior to traditional polymer and silica membranes^23^. Ambrosetti and Silvestrelli designed two types of graphene with hydrogen saturated pores and examined the permeation barriers of several gas molecules with ab initio simulations^24^. Zhang *et al*. proposed a convenient method for the gas separation based on the kinked single-walled CNTs with adjustable permeability by the bending angle of CNTs^25^. Liu *et al*. revealed a highly efficient separation for removing CO_2_ from natural gas through a windowed carbon nanotubes^26^. Han *et al*. synthesized a composite membrane via placing a selective layer on a polyethersulfone nanoporous substrate, whose CO_2_ permeance is enhanced through dispersing multi-walled CNTs wrapped by a copolymer poly^27^. These previous investigations have demonstrated that the tradeoff between the permeability and selectivity could be improved with the use of nanoscale carbon materials. However, for a certain type of membranes fabricated by the corresponding technology, in general, the permeability and selectivity are always fixed and hardly changed. Hence, it has to choose the appropriate membranes according to the mixtures and requirements. This is rather inconvenient especially for the separation of the multicomponent mixture.

Recently, the fluid-filled CNTs have been successfully fabricated and extracted in laboratories through the density gradient ultracentrifugation^8,9^, the laser irradiation^28^, the exposure to water vapor^29^ and the soaking in dilute salt solution^30^. As compared to the empty CNTs, the water-filled and salt water-filled CNTs could make a deformation response to the external electric field^31,32^. Consequently, the membrane composed of the fluid-filled CNTs should possess controllable permeability and selectivity through changing the electric field, which provides a feasible way for the adjustable separation of the multicomponent mixture. In this paper, we construct a salt water-filled CNT membrane for gas separation. The characteristic size of the effective molecular channel under the electric field is estimated with a simplified computational model. The molecular dynamics (MD) simulation is performed to examine the separation process of the binary and three-component gas mixture, respectively. This work provides a promising route for the application of the novel fluid-filled CNTs in the design and fabrication of the adjustable separation membrane.

## Results

### Membrane characterization

**Figure 1:**
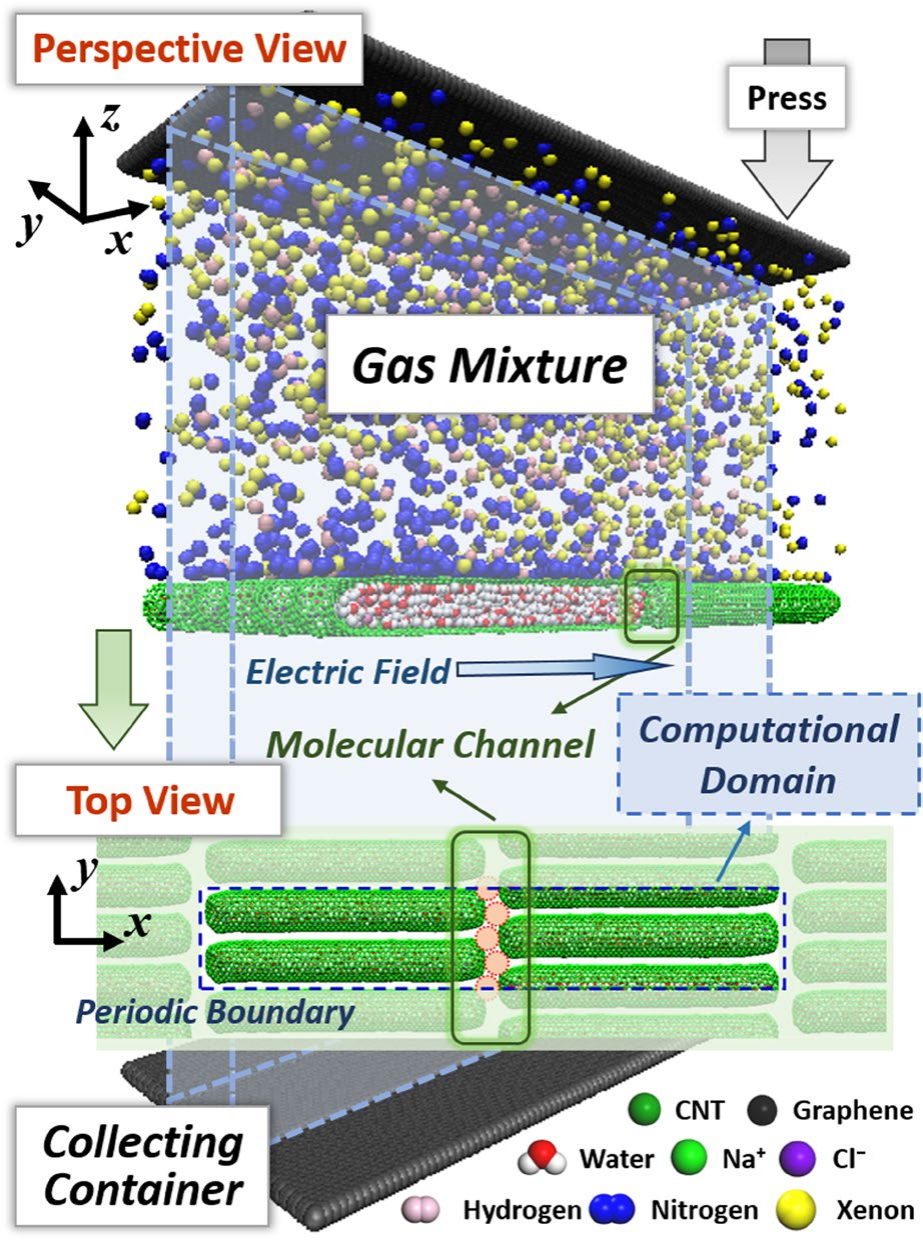
Schematic illustration of the salt water-filled CNT membrane to separate the gas mixture of the hydrogen, nitrogen and xenon, which are labelled as the pink, blue and yellow balls, respectively. The lower inset depicts the top view of the separation membrane from the z axis, where the internal red circular region shows the effective area of the molecular channel. The blue dashed framework and rectangle in the inset indicate the computational domain with periodic boundary in this work.

By means of the salt water-filled CNTs, a membrane with adjustable molecular channel is proposed for the separation of gas mixture. As shown in Fig. 1, the separation membrane is composed of the salt water-filled CNTs with the parallel arrangement along the *y* direction and the staggered arrangement along the *x* direction. The previous researches have revealed that the fluid-filled CNTs with different diameters exhibit different tension deformations^31,32^. Here, we adopt the (12, 12) CNTs with the length of about 130 Å to construct the membrane. The concentration of the encapsulated salt water inside the capped CNTs is about 3.4 wt.%, which is comparable to that of sea water. The initial lateral distance along the *y* direction between the adjacent CNTs is 3.72 Å. This distance is so narrow that the general gas molecules cannot pass through. The gaps between the ends of the CNTs are responsible for the transport of gas molecules. The initial gap between the CNT ends in this study is about 7.5 Å. For the practical materials, the initial lateral distances and channel gaps can be adjusted by decorating the CNT walls, and can be further changed through stretching or compressing the membrane. The effective area of the molecular channel for gas transport is approximately regarded as a circle here as labelled in Fig. 1, whose characteristic size can be changed by applying an axial electric field. This is because that the electric field forces stemming from the internal ions and polar water molecules result in the tensile deformation of CNTs^31,32^. Thus, the mixed gases with different effective molecular sizes can be separated by selecting an appropriate intensity of the electric field. Especially for a multicomponent gas mixture, the proposed CNTs membrane could separate and purify the mixed gases in turn only through changing the intensity of electric field without changing the separation membrane.

### Separation mechanism

The present separation strategy based on the proposed salt water-filled CNT membrane belongs to the physical separation. Under the specified electric field, the gaps between the CNT ends provide a molecular channel with a corresponding characteristic size for gas diffusion. Only the gas molecules with the effective diameter smaller than the characteristic size of molecular channels could pass through the membrane, and the large gas molecules will be prevented in the upper container. Hence, the critical evaluation for the gas separation is to estimate the characteristic size of the molecular channel and the effective diameter of gas molecules.

#### The characteristic size of the molecular channel

**Figure 2:**
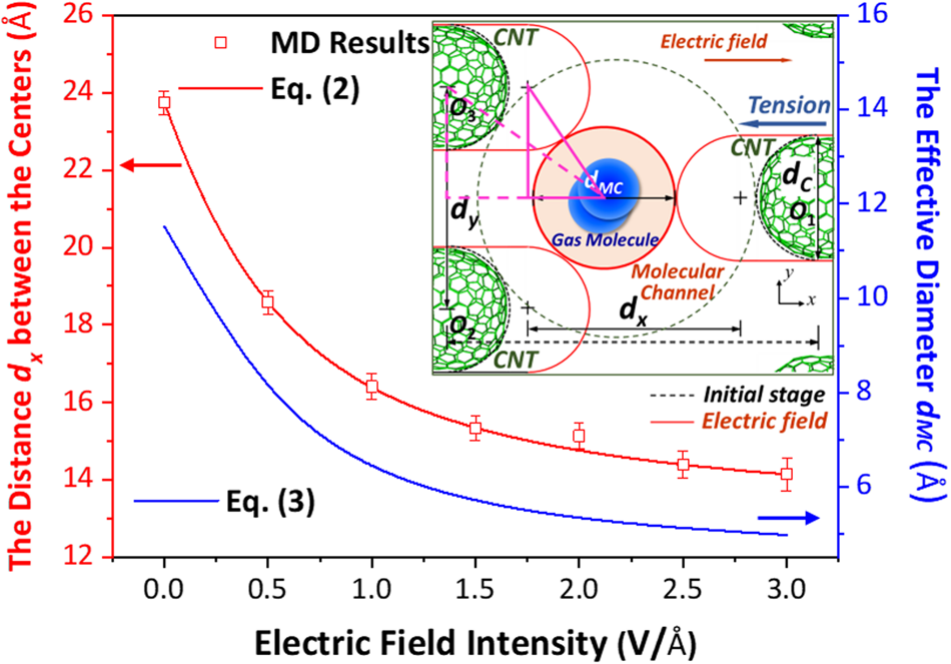
The variation of the distance *d*_*x*_ and the effective diameter *d*_*MC*_ with the electric field intensity. The inset shows the simplified computational model of the molecular channel. The black dashed lines indicate the profiles of the CNT ends and the molecular channel in the initial state. The red lines represent the corresponding profiles under electric field. The pink lines are the auxiliary triangles for obtaining Eq. (1).

The inset in Fig. 2 shows the simplified computational model to estimate the characteristic size of the molecular channel. The ends of the capped CNTs are approximately considered as semicircles. The common tangent circle to the CNT ends labelled as red circle is seen as the effective molecular channel for gas transport. According to the illustrated geometrical relationship, the diameter of the effective circular area *d*_*MC*_ can be expressed as

1 $${d}_{MC}=\frac{{{d}_{y}}^{2}}{4{d}_{x}}+{d}_{x}-{d}_{C}$$

where *d*_*C*_ = 16.28 Å is the diameter of (12, 12) CNTs, *d*_*x*_ and *d*_*y*_ = 20 Å are the center distances between the adjacent CNTs along the *x* and *y* directions, respectively. The distance *d*_*x*_ will vary with the electric field intensity, as shown in Fig. 2. According to the simulation results, the relationship between *d*_*x*_ and the electric field intensity *E* (0 ~ 3.0 V/Å) could be fitted as:

2 $${d}_{x}=\sqrt{{(aE)}^{2}+b}-aE+12.85$$

where *a* = 13.62 Å/V and *b* = 114.5 Å^2^ are the fitting results according to the MD simulations on the equilibrium state of the salt water-filled CNTs under electric fields. The corresponding MD models and settings are consistent with the membrane for the gas separation. The form of Eq. (2) is derived with a simple assumption that the repulsive force is in inverse proportion to the distance between the CNT ends along the *x* direction. Actually, the repulsive force here mainly includes the van der Waals force (calculated by LJ potential) and the Coulomb force, which are all in inverse proportion to the power of distance. Based on the simple assumption, the form of the Eq. (2) is founded and the corresponding error is corrected by the two fitting parameters. The results indicate that the average error between the MD results and Eq. (2) is about 1.0% within the range of 0 ~ 3 V/Å. Moreover, when *E* → ∞, *d*_*x*_ → 12.85 Å, which is the limiting distance of the two adjacent CNT ends along the *x* direction. According to Eqs (1) and (2), the relationship between the characteristic size *d*_*MC*_ of the effective molecular channel and the electric field intensity *E* can be written as

3 $${d}_{MC}=\frac{100}{\sqrt{{(aE)}^{2}+b}-aE+12.85}+\sqrt{{(aE)}^{2}+b}-aE-3.43$$

The corresponding results are also plotted in Fig. 2.

#### The effective diameter of gas molecules

**Figure 3:**
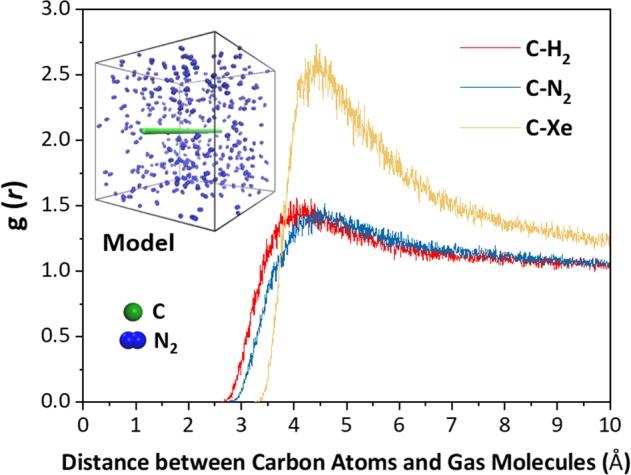
The radial distribution functions of the three gas molecules to the carbon atoms. The inset shows the computational model of the carbon chain and N_2_. The green and blue balls illustrate the carbon and nitrogen atoms, respectively.

In this work, three simple gases H_2_, N_2_ and Xe are adopted to make up the gas mixture, whose interactions are simulated by the LJ potentials. To obtain an accurate estimation on the effective diameters of the three gas molecules, the numerical experiments of a carbon chain immersed in the pure gases is conducted, respectively, as shown in the inset of Fig. 3. The interval of the carbon atoms in the chain is 1.42 Å, which is identical to the bond length of CNTs.

Figure 3 gives the radial distribution functions g(*r*) of the three gas molecules to the carbon atoms. The smallest distance for g(*r*) > 0 implies the limiting reachable position of the gas molecules to the carbon atoms, but there is merely little chance for finding the gas molecules within this distance. Here, the double distances corresponding to g(*r*) > 0.02 are deemed as the overall effective diameters of the three gas molecules, as listed in Table 1. In fact, compared to the mentioned smallest distances, the adopted distances are slightly larger by 0.10 Å in average, which can still be approximately seen as the smallest diameters and is rather critical for the tradeoff between the separation efficiency and purity.

**Table 1:** The overall effective diameter of the three gas molecules to carbon atoms.

Gas	H_2_	N_2_	Xe
*d*_*OG*_ (Å)	5.32	5.75	6.60

### Separation of the binary gas mixture

The gas mixture of the hydrogen (H_2_) and nitrogen (N_2_) is employed to examine the separation efficiency of the binary gas mixture based on the salt water-filled CNT membrane. In this separation, the volumes of the upper and lower containers are fixed. No external pressure is applied and the separation process only depends on the molecular free diffusion driven by the concentration difference. Initially, the molecular amount of each gas in the upper container is 412. Four intensities of the axial electric field varying from 0.0 to 3.0 V/Å are considered here, respectively. As the electric field intensity increases, the effective diameter of molecular channel decreases from 11.52 Å to 4.98 Å, which can be calculated by Eq. (3). The overall effective diameters *d*_*OG*_ of H_2_ and N_2_ molecules are 5.32 Å and 5.75 Å, respectively.

**Figure 4:**
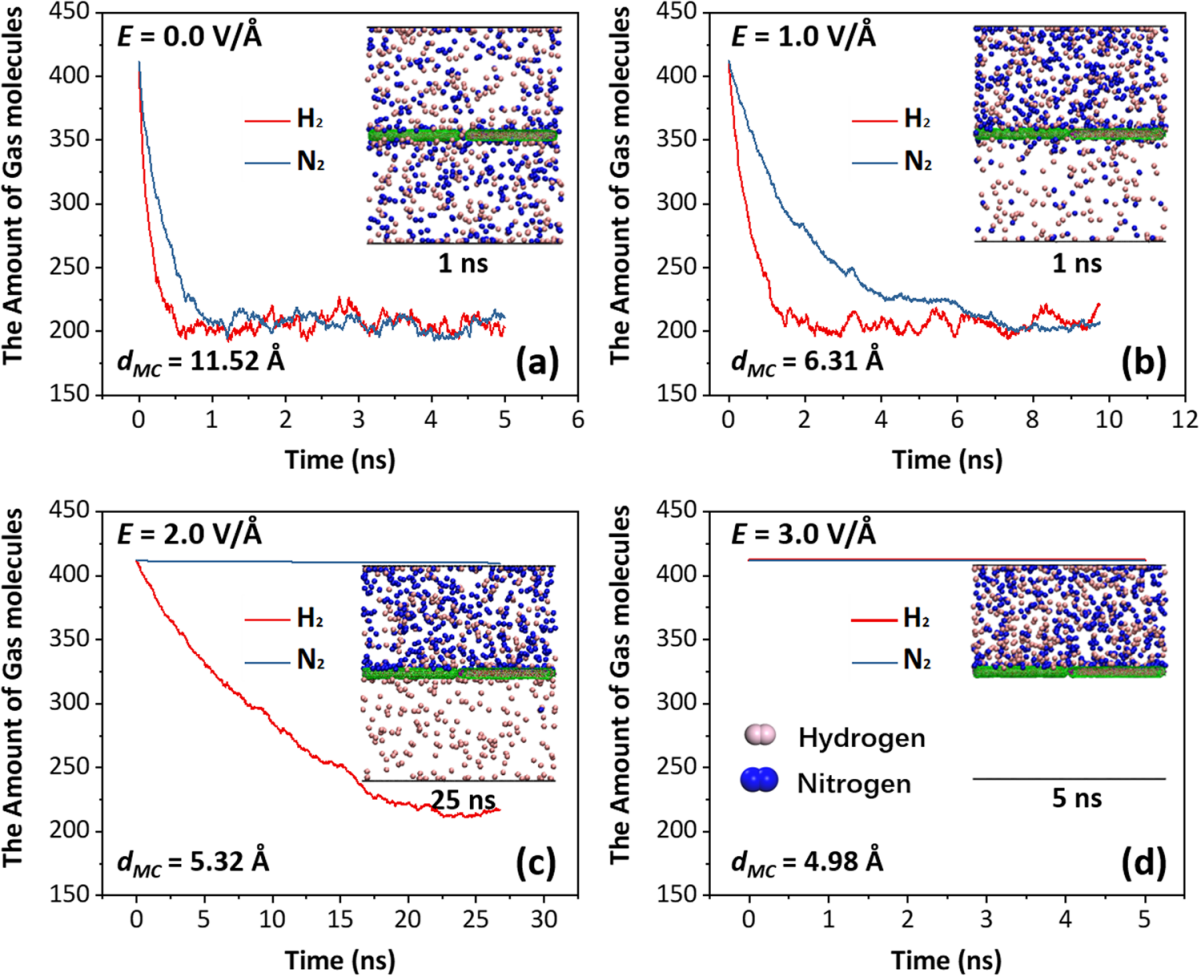
The amounts of the unseparated hydrogen and nitrogen molecules in the upper container with the time. (**a**–**d**) Correspond to the separation processes under the electric field of the four intensities increasing from 0.0 to 3.0 V/Å, respectively. The insets depict the instantaneous distributions of the gas molecules at the specific time.

Figure 4 shows the variations of the amount of the two gas molecules in the upper container with the time. When no electric field is applied, the characteristic size of the molecular channel is obviously larger than the overall effective diameters of the H_2_ and N_2_ molecules. Hence, it can be seen from Fig. 4(a) that both of the two gas molecules could rapidly pass through the membrane and the equilibrium state is achieved at about 1 ns. During the initial transport process, the hydrogen possesses a slightly higher permeation rate to the saturation relative to the nitrogen because of its smaller effective diameter. The average membrane permeabilities in 1 ns are about 4.22 and 3.94 × 10^4^ mol/m^2^s for the hydrogen and nitrogen, respectively. Here, the membrane permeability is calculated by the moles of the permeated gas molecules to the pore area and time^22^. When the electric field intensity increases to 1.0 V/Å (Fig. 4(b)), the characteristic size of the molecular channel decreases to 6.31 Å, which is still larger than the overall effective diameters of H_2_ and N_2_ molecules. Therefore, the two gas molecules could still pass through the membrane. But the permeation rate has a remarkable decrease due to the shrinking characteristic size of molecular channel, especially for the nitrogen. Here, as the pore area decreases, the average membrane permeabilities in 1 ns increase to about 11.35 and 5.71 × 10^4^ mol/m^2^s for the hydrogen and nitrogen, respectively. In 1 ns, the ratio of the amount of the H_2_ molecules to that of the N_2_ molecules is 1.99. The amount of the gas molecules in the lower container under 1.0 V/Å is indeed less than that without electric field at 1 ns, as shown in the insets of Fig. 4(a,b). The total equilibrium time to saturation is about 7 ns. As the electric field intensity further increases to 2.0 V/Å, the characteristic size of the molecular channel is about 5.32 Å, which is comparable to the overall effective diameter of H_2_ molecule but smaller than that of N_2_ molecule. Hence, the hydrogen molecules could pass through the membrane whereas the nitrogen molecules hardly permeate. Thus, the salt water-filled CNT membrane could separate the gas mixture of the hydrogen and nitrogen under the electric field of 2.0 V/Å, as shown in Fig. 4(c). In 1 ns, the average membrane permeability of the hydrogen is about 1.77 × 10^4^ mol/m^2^s, and no nitrogen molecule is observed to pass through the membrane. The final purification rate reaches up to 99.03%. The permeation of several nitrogen molecules is attributed to the thermal vibration of CNT ends. When the intensity of electric field is 3.0 V/Å, the characteristic size of the molecular channel is only 4.98 Å, which is smaller than the overall effective diameters of the hydrogen and nitrogen molecules. It can be observed from Fig. 4(d) that the membrane is totally closed and no gas molecules could pass through the present channel. The simulation results suggest that the proposed salt water-filled CNT membrane could control the flow rate and even precisely separate the gas mixture by means of the electric field.

### Separation of the gas mixture with three components

The present separation membrane has a significant advantage for the multicomponent gas mixture. The gas separation can be achieved one by one through adjusting the electric field intensity. Here, the gas mixture of the hydrogen (H_2_), nitrogen (N_2_) and xenon (Xe) is considered to clarify the separation process and examine the separation efficiency. The overall effective diameters of the hydrogen, nitrogen and xenon molecules increase in turn (Table 1). Thus, the separation process can be divided into two stages. The first stage is to gather the hydrogen with the smallest overall effective diameter and the second stage is to collect the nitrogen with the second-smallest overall effective diameter. The electric field intensities in these two stages are determined according to the overall effective diameters of the gases to be separated on the basis of Eq. (3). Generally, the characteristic size of the molecular channel should fall around the effective diameters of the present and next gases to be separated, and therefore the electric field intensity is always within a range. The weaker electric field intensity (larger characteristic size) could enhance the separation velocity but reduce the separation purity. On the contrary, the largest available electric field intensity (smallest characteristic size) could obtain more pure separated gas but the separation velocity will become slow. Hence, the appropriate electric field intensity should be a tradeoff according to the separation requirement. In this separation, the external pressure is considered through moving down the upper graphene wall gradually to the height corresponding to the required volume of the unseparated gases. Moreover, the separated gas through the separation membrane to the lower container will be collected every 50 ps.

**Figure 5:**
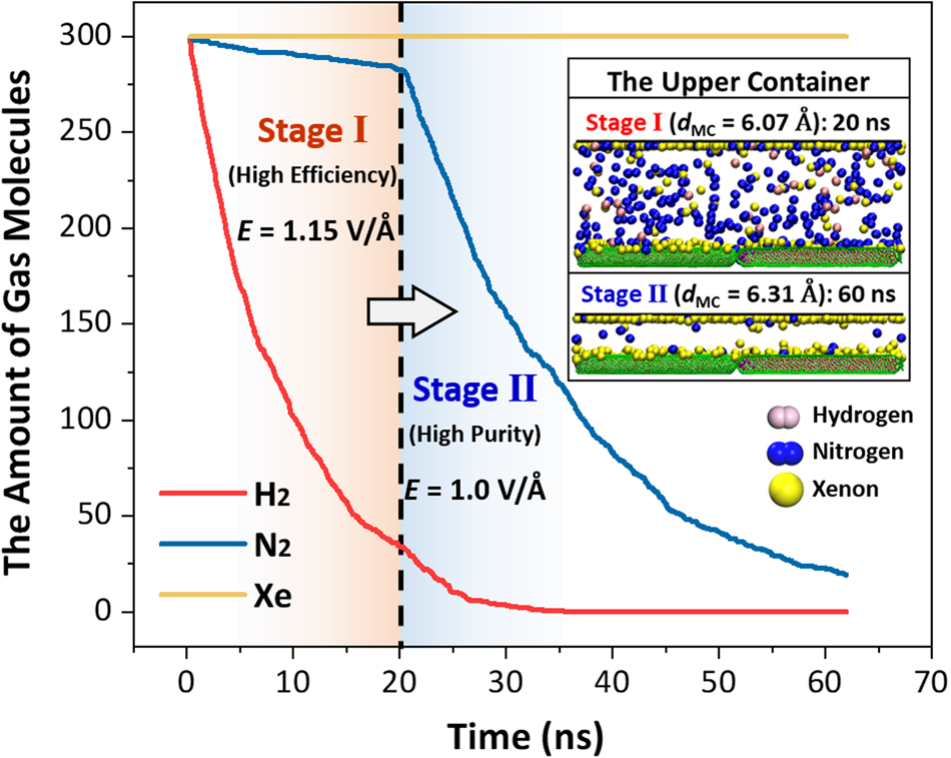
The amounts of the unseparated hydrogen, nitrogen and xenon molecules in the upper container with the time. The dashed line indicates the transition time of the two separation stages. The inset shows the instantaneous distributions of the gas molecules in the upper containers at 10 ns (stage I) and 60 ns (stage II).

At the stage (I), to obtain a high permeation rate, we choose a small electric field intensity 1.15 V/Å, which corresponds to the large characteristic size of the molecular channel (~6.07 Å). This characteristic size is distinctly larger than the overall effective diameter of the hydrogen molecule but slightly larger than that of the nitrogen molecule. Thus, H_2_ has a high permeation rate, but N_2_ could only squeeze through the narrow channel and Xe cannot pass through the separation membrane (Fig. 5). At 20 ns, the average membrane permeabilities of the hydrogen and nitrogen are about 9.54 and 0.62 × 10^3^ mol/m^2^s, respectively, and 88.7% of H_2_, 5.8% of N_2_ and no xenon molecules in the upper container have permeated into the lower collection. Compared to the separation of the binary gas mixture in the Section 3.2 under the electric field with the intensity of 2.0 V/Å, the separation rate has a promotion but the separation purity slightly decreases.

Subsequently, the electric field intensity is reduced to 1.0 V/Å and the separation process moves into the stage (II). In this stage, the emphasis is placed on the separation purity. Hence, the characteristic size of the molecular channel under the adopted electric field intensity (6.31 Å) is slightly smaller than the overall effective diameter of the xenon molecule. Thus, the xenon can be resisted by the separation membrane. During 20 ~ 40 ns, the average membrane permeability of the nitrogen is about 6.0 × 10^3^ mol/m^2^s. As shown in the inset of Fig. 5, most of the nitrogen molecules have permeated into the lower container and only about 7.6% still stay in the upper container at 60 ns. During the separation process, no xenon molecules pass through the membrane. The present simulation demonstrates the convenient separation of the multicomponent gas mixture based on the salt water-filled CNTs. The separation efficiency and purity can even be adjusted in terms of the product demand by the electric field intensity.

## Discussion

In this work, a controllable separation membrane is designed on the basis of the salt water-filled CNTs. The characteristic size of the molecular channel is changeable through adjusting the electric field intensity. The corresponding controlling relationship is established by means of a simplified theoretical model, which is utilized to separate the mixed gas whose effective diameters are calculated through the radial distribution function. To examine the feasibility and validity of the proposed separation membrane, the numerical experiment via MD simulation is performed to observe the separation processes of the gas mixture with the two and three components, respectively. The simulation results of the binary gas mixture (H_2_ and N_2_) exhibit the diverse permeation behaviors under the electric field of the four intensities, which indicate that the control of the flow rate and gas separation can be achieved by adjusting the electric field. As for the separation of the gas mixture with the three components (H_2_, N_2_ and Xe), the high-efficiency separation and high-purity separation are explored, respectively, through choosing the electric field intensity according to the corresponding requirements. The simulation results indicate that the successive separation of the multicomponent gas mixture could be achieved on the basis of the same membrane in combination with the electric field of different intensities. The reported research findings demonstrate a novel separation or purification strategy for the multicomponent mixture. Moreover, with the fluid-filled CNTs, which have been successfully fabricated in laboratory, the proposed procedure not only provides a potential application on the mixture separation but also has an insight into the design of the nanoscale controllable equipment such as nanoscale probe, sensor, switch and so on.

## Methods

To verify the feasibility of the proposed separation membrane for the gas separation, we construct a numerical model and the MD method is adopted to examine the separating process. Here, the MD simulations are conducted with the open-source software LAMMPS^33^, whose validity on the mechanical property of solid materials^34,35^ and the gas separation has been demonstrated by the previous works^36,37^. The simulations are carried out in the cuboid box (273.0 Å × 39.9 Å × 320.0 Å, the blue framework in Fig. 1) with the periodic boundary conditions in the *x* and *y* directions. The atomic interactions of carbon atoms are described by the reactive empirical bond order (REBO) potential^38^, which has been used successfully in many previous studies on the calculations of mechanical properties and deformation behaviours of CNTs^39,40^. The middle parts of the CNTs are fixed whereas the ends are free. The interactions between the atoms from the different molecules are calculated with the Lennard–Jones (LJ) and the Coulomb electrostatic potentials. The detailed LJ parameters are listed in Tables S1 and S2 in the Supplementary Information. The water is simulated using the TIP4P-EW model^41^, which could well capture the intermolecular and intramolecular polarizations of water^42,43^. The SHAKE algorithm is used to enable the water molecules to maintain their intrinsic geometrical configurations, i.e., the bond length of 0.9572 Å and the angle degree of 104.52°, respectively. The simulation is carried out within the NVT ensemble at a temperature around 298 K, which is controlled by the Nosé-Hoover thermostat. The initial relaxation time is 0.2 ns with a time step of 1 fs. In this stage, the gas mixture is confined in the upper container by two parallel graphenes. Subsequently, an electric field along the *x* direction is applied to induce the tensile deformation of the salt water-filled CNTs. The characteristic size of the molecular channel is therefore changed and becomes stable after the equilibrium time of 0.1 ns. Finally, the upper container is opened through removing the lower graphene wall, and the molecular channel is exposed to the gas molecules for separation. For the multicomponent gas mixture, the separation process can be divided into the several stages through adjusting the electric field intensity according to the effective diameters of different mixed gases. All the simulation results are the averages of three independent simulations to eliminate the random effects of the initial velocity and configuration.

## Supplementary information

An adjustable permeation membrane up to the separation for multicomponent gas mixture

## Data Availability

The datasets generated and analysed during the current study are available from the corresponding author on reasonable request.
