# Supplementary material for: An adjustable permeation membrane up to the separation for multicomponent gas mixture
Source: Sci Rep. 2019 May 14;9:7380. doi: 10.1038/s41598-019-43751-0 (PMC6517568; doi:10.1038/s41598-019-43751-0)
Supplement: Supplementary file 1 — An adjustable permeation membrane up to the separation for multicomponent gas mixture [file 41598_2019_43751_MOESM1_ESM.pdf]

# Supplementary information for:

## An adjustable permeation membrane up to the separation for multicomponent gas mixture

Hongfei Ye[1], Dong Li[1], Xin Ye[1], Yonggang Zheng[1], Zhongqiang Zhang[2], Hongwu Zhang[1,*] & Zhen Chen[1,3,*]

[1] International Research Center for Computational Mechanics, State Key Laboratory of Structural Analysis for Industrial Equipment, Department of Engineering Mechanics, Faculty of Vehicle Engineering and Mechanics, Dalian University of Technology, Dalian 116024, P. R. China

[2] Micro/Nano Science and Technology Center, Jiangsu University, Zhenjiang 210013, P.R. China

[3] Department of Civil and Environmental Engineering, University of Missouri, Columbia, MO 65211, USA

*zhanghw@dlut.edu.cn and ChenZh@missouri.edu

### The LJ potential parameters in the MD simulations

Table S1. LJ Parameter $\sigma$

| $\sigma$ (Å) | $C_1$ | O | $H_1$ | Na | Cl | Xe | N | $C_2$ | $H_2$ |
|---|---|---|---|---|---|---|---|---|---|
| $C_1$ | 3.40 [1] | 3.28 | 0.00 | 2.88 | 3.90 | 3.75 | 3.36 | 0.00 | 3.18 |
| O | - | 3.16 [2] | 0.00 | 2.76 | 3.78 | 3.63 | 3.24 | 0.00 | 3.06 |
| $H_1$ | - | - | 0.00 [2] | 0.00 | 0.00 | 0.00 | 0.00 | 0.00 | 0.00 |
| Na | - | - | - | 2.35 [3] | 3.38 | 3.23 | 2.84 | 0.00 | 2.66 |
| Cl | - | - | - | - | 4.40 [3] | 4.25 | 3.86 | 0.00 | 3.68 |
| Xe | - | - | - | - | - | 4.10 [4] | 3.71 | 3.75 | 3.53 |
| N | - | - | - | - | - | - | 3.32 [5] | 3.36 | 3.14 |
| $C_2$ | - | - | - | - | - | - | - | 3.40 [2] | 3.18 |
| $H_2$ | - | - | - | - | - | - | - | - | 2.96 [5] |

Table S2. LJ Parameter $\varepsilon$

| $\varepsilon$ (eV) | $C_1$ | O | $H_1$ | Na | Cl | Xe | N | $C_2$ | $H_2$ |
|---|---|---|---|---|---|---|---|---|---|
| $C_1$ | 0.00373 [1] | 0.00513 | 0.00000 | 0.00459 | 0.00403 | 0.00842 | 0.00342 | 0.00000 | 0.00332 |
| O | - | 0.00706 [2] | 0.00000 | 0.00631 | 0.00554 | 0.01158 | 0.00471 | 0.00000 | 0.00456 |
| $H_1$ | - | - | 0.00000 [2] | 0.00000 | 0.00000 | 0.00000 | 0.00000 | 0.00000 | 0.00000 |
| Na | - | - | - | 0.00565 [3] | 0.00496 | 0.01036 | 0.00421 | 0.00000 | 0.00408 |
| Cl | - | - | - | - | 0.00435 [3] | 0.00909 | 0.00370 | 0.00000 | 0.00358 |
| Xe | - | - | - | - | - | 0.01900 [4] | 0.00772 | 0.00842 | 0.00749 |
| N | - | - | - | - | - | - | 0.00314 [5] | 0.00342 | 0.00304 |
| $C_2$ | - | - | - | - | - | - | - | 0.00373 [1] | 0.00332 |
| $H_2$ | - | - | - | - | - | - | - | - | 0.00295 [5] |

Here, $C_1$ and $C_2$ represent the carbon atoms in the CNT and graphene, respectively. $H_1$ and $H_2$ are the hydrogen atoms in the water molecule ($H_20$) and hydrogen molecule ($H_2$), respectively. The LJ parameters between the different atoms are calculated according to the Lorentz-Berthelot mixing rules[6].

## Reference


1. Hummer, G., Rasaiah, J.C. & Noworyta, J.P. Water conduction through the hydrophobic channel of a carbon nanotube. *Nature* **414**, 188-190 (2001). DOI: 10.1038/35102535
2. Horn, H.W. *et al*. Development of an improved four-site water model for biomolecular simulations: TIP4P-EW. *J. Chem. Phys*. **120**, 9665-9678 (2004). DOI: 10.1063/1.1683075
3. Qiao, R. & Aluru, N.R. Atypical Dependence of electroosmotic transport on surface charge in a single-wall carbon nanotube. *Nano Lett.* **3**(8), 1013-1017 (2003). DOI: 10.1021/nl034236n
4. Santikary, P., Yashonath, S. & Ananthakrishna, G. A molecular dynamics study of xenon sorbed in sodium Y zeolite. 1. Temperature and concentration dependence. *J. Phys. Chem.* **96**, 10469-10477 (1992). DOI: 10.1021/j100204a065
5. Du, H.L. *et al*. Separation of hydrogen and nitrogen gases with porous graphene membrane. *J. Phys. Chem. C* **115**, 23261-23266 (2011). DOI: 10.1021/jp206258u
6. Delhommelle, J. & Millié, P. Inadequacy of the Lorentz-Berthelot combining rules for accurate predictions of equilibrium properties by molecular simulation. *Mol. Phys.* **99**(8), 619-625 (2001). DOI: 10.1080/00268970010020041
